# MarkerGenie: an NLP-enabled text-mining system for biomedical entity relation extraction

**DOI:** 10.1093/bioadv/vbac035

**Published:** 2022-05-13

**Authors:** Wenhao Gu, Xiao Yang, Minhao Yang, Kun Han, Wenying Pan, Zexuan Zhu

**Affiliations:** 1 College of Computer Science and Software Engineering, Shenzhen University, Shenzhen 518060, China; 2 GeneGenieDx Corp, San Jose, CA 95134, USA

## Abstract

**Motivation:**

Natural language processing (NLP) tasks aim to convert unstructured text data (e.g. articles or dialogues) to structured information. In recent years, we have witnessed fundamental advances of NLP technique, which has been widely used in many applications such as financial text mining, news recommendation and machine translation. However, its application in the biomedical space remains challenging due to a lack of labeled data, ambiguities and inconsistencies of biological terminology. In biomedical marker discovery studies, tools that rely on NLP models to automatically and accurately extract relations of biomedical entities are valuable as they can provide a more thorough survey of all available literature, hence providing a less biased result compared to manual curation. In addition, the fast speed of machine reader helps quickly orient research and development.

**Results:**

To address the aforementioned needs, we developed automatic training data labeling, rule-based biological terminology cleaning and a more accurate NLP model for binary associative and multi-relation prediction into the *MarkerGenie* program. We demonstrated the effectiveness of the proposed methods in identifying relations between biomedical entities on various benchmark datasets and case studies.

**Availability and implementation:**

MarkerGenie is available at https://www.genegeniedx.com/markergenie/. Data for model training and evaluation, term lists of biomedical entities, details of the case studies and all trained models are provided at https://drive.google.com/drive/folders/14RypiIfIr3W_K-mNIAx9BNtObHSZoAyn?usp=sharing.

**Supplementary information:**

Supplementary data are available at *Bioinformatics Advances* online.

## 1 Introduction

Relations of biomedical entities (bioentities) are critical to biomedical studies and are hidden in a large number of biomedical articles. In this work, the main goal is to rapidly and accurately identify *associative* relations between a pair of biomedical entities present in the literature. We consider two entities to be *associative* in a context when they are described to be correlated directly, causal or non-causal. Most biomedical entity relations such as a biomarker and a disease are associative. Determining such a relation is typically an important first step to guide additional wet-lab or clinical studies to verify the *diagnostic, predictive, prognostic, predisposing* and *treatment* relation. Without being exhaustive, a bioentity may refer to a disease, a gene, a metabolite or a microbial taxa.

Many biomedical text-mining methods have been used to identify associations of diseases and biomarkers. These methods can digest research articles more efficiently and comprehensively compared to human researchers and can help prioritize the targets in diagnoses and drug target discovery. In the following, we provide a brief overview of the current status of biomarker relation database curation and text-mining methods.

Bioentity relation databases are typically manually curated and serve as the ground truth for the research community. For example, Ma *et al.* (2017) manually extracted 292 microbe–microbe, 39 disease–disease and 483 microbe–disease associations from microbiome-related articles. Janssens *et al.* (2018) established a disease–microbiome database by querying PUBMED database using criteria ([(’microbiota’ OR ’microbiome) and (’health’ OR ’disease’)] and [microbiome alterations]). Then, the disease, microbiome terms and their relations were extracted manually. Noronha *et al.* (2019) created a database that relates human metabolism with genetics, microbial metabolism, nutrition and diseases.

To automate and expand the scope of entity relation extraction, a few methods, including PolySearch2 (Liu *et al.*, 2015), BEST (Lee *et al.*, 2016), GenCLiP 3 (Wang *et al.*, 2020), STRING (Szklarczyk *et al.*, 2021), IBDDB (Khan *et al.*, 2021) and DrugShot (Kropiwnicki *et al.*, 2022), have been introduced. With a common assumption that the strength of entity association is positively correlated with their co-occurring frequency in the same context, these methods first identified frequently co-occurring entities of interest, then refined the entity relation with different scoring and filtering criteria. However, there are some limitations. A pair of entities with low co-occurring frequency can be reliable but would be missed. For example, recently discovered relations would have few mentions in the literature. Meanwhile, high co-occurrence counts can include many false positives that require *ad hoc* and complex rules to eliminate.

These limitations have been addressed by supervised machine learning (ML)-based methods (Hsieh *et al.*, 2017; Hua and Quan, 2016; Xu *et al.*, 2015). To curate CIViC database (Lever *et al.*, 2019), published literature was parsed and sentences containing a pair of target entities were identified via exact string matching. A support vector machine-based classifier was then trained using 800 labeled sentences. Ahmed *et al.* (2019) proposed a novel neural network architecture for identifying protein–protein interactions (PPIs) from biomedical text using a tree long-short-term memory (LSTM) network with structured attention to traverse the dependency tree of a sentence through a child sum tree LSTM. Meanwhile, structural information was learned through a parent selection mechanism by modeling non-projective dependency trees. The main challenge for the application of ML methods is the lack of labeled training data. Although distant supervision (Mintz *et al.*, 2009) can be used to acquire additional training data with positive labels, negative training data cannot be generated and this method requires a high-quality knowledge database that is typically hard to curate.

In this article, we treat finding relevant biomedical entities as a sentence-level binary/multiple relation classification task. During entity extraction, we introduced rule-based strategies to reduce false positive extractions as the existing bioentity terminologies still contain a large number of ambiguities and sometimes, errors. To address the lack of training data and the labor-intensive manual labeling process, we proposed an automated training data generation using co-occurrence frequency matrix and demonstrated its practical use. We then developed a new model, SBGT (SciBERT+Gumbel Tree-GRU), for relation classification that uses SciBERT (Beltagy *et al.*, 2019) to encode the context features of words and Gumbel Tree-GRU (Hong *et al.*, 2020) to encode the syntactic structures of sentences.

**Fig. 1.**
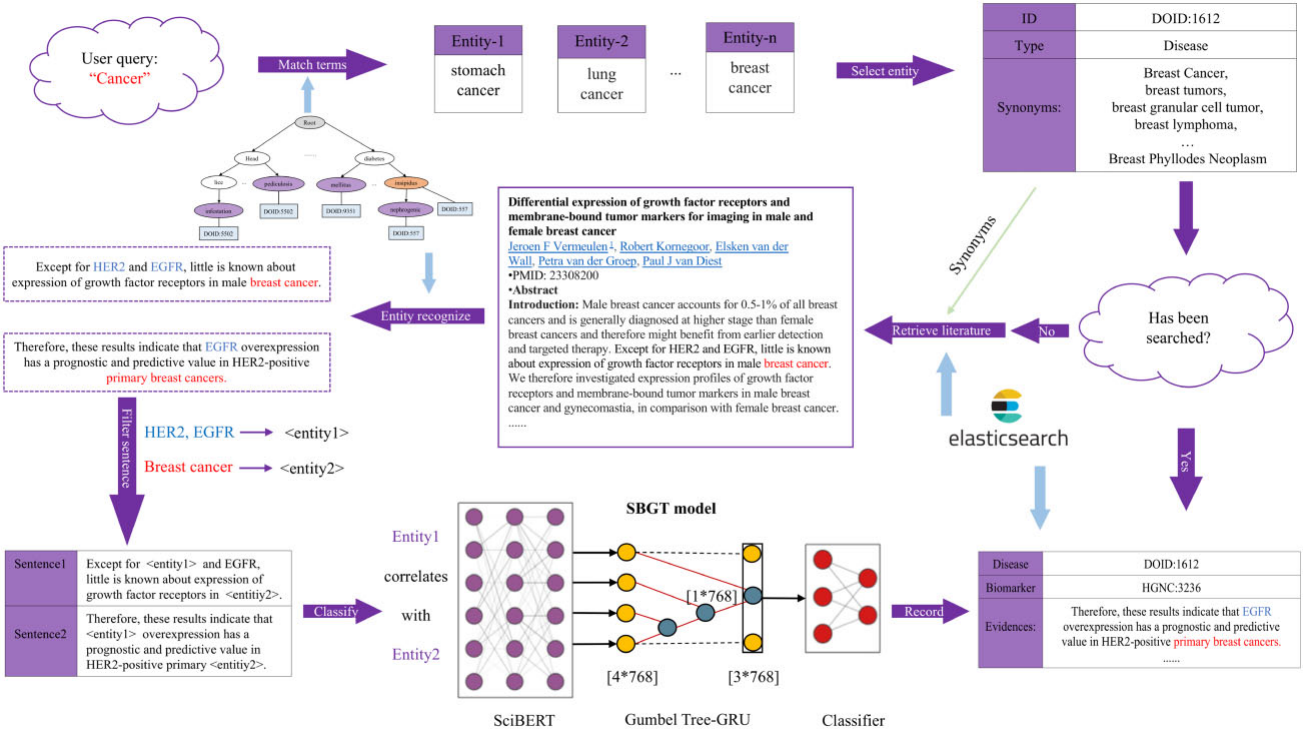
MarkerGenie online workflow. MarkerGenie is a text-mining system for identifying biomarker relations with diseases. Given a query disease term, MarkerGenie first identifies relevant disease terms through fuzzy matching. Then, it retrieves articles according to the synonyms of the disease, and select the sentences that contain both the disease and biomarkers through entity extraction. Afterwards, the filtered sentences are classified by NLP models. Finally, the system returns the biomarkers related to the disease extracted from the literature in detail that including the source sentences, tables and articles. To improve speed, result caching was used

We provide MarkerGenie as an online text-mining tool. The current release includes the following entities: diseases, microbiomes, genes and metabolites. The corpus currently includes the free-text and tables of articles in PubMed and PubMed Central. The overview of MarkerGenie is given in Figure 1 that includes four main components: user query processing, article retrieving and sentence filtering, model-based classification and results reporting. The implementation details of MarkerGenie are provided in Supplementary Section S1.

## 2 Materials and methods

### 2.1 SBGT model

In the proposed SBGT model, we used SciBERT (Beltagy *et al.*, 2019) to extract the contextual features of words given the input sentence. SciBERT can improve the handling of unseen and rare words by using subword tokenizer in between words and characters. It had been experimentally shown to outperform BERT-Base and Bio-BERT in relation extraction of biomedical text (Beltagy *et al.*, 2019). Then, we used the Gumbel Tree-GRU (Hong *et al.*, 2020) to encode the syntactic structure. The encoded vectors were concatenated and fed into a fully connected layer for prediction. As shown in Figure 1, given a sentence, SciBERT extracts the contextual features of each word. Each word is encoded as a 1*768 vector. Then Gumbel Tree-GRU is used to organize those words into a vector to represent the sentence. Afterward, the vector as well as the contextual features of Entity1 and Entity2 are concatenated to indicate their relation. Finally, a fully connected layer is applied to predict the probability of the relation falling within each category.

### 2.2 Unsupervised training data generation for binary relation classification

**Fig. 2.**
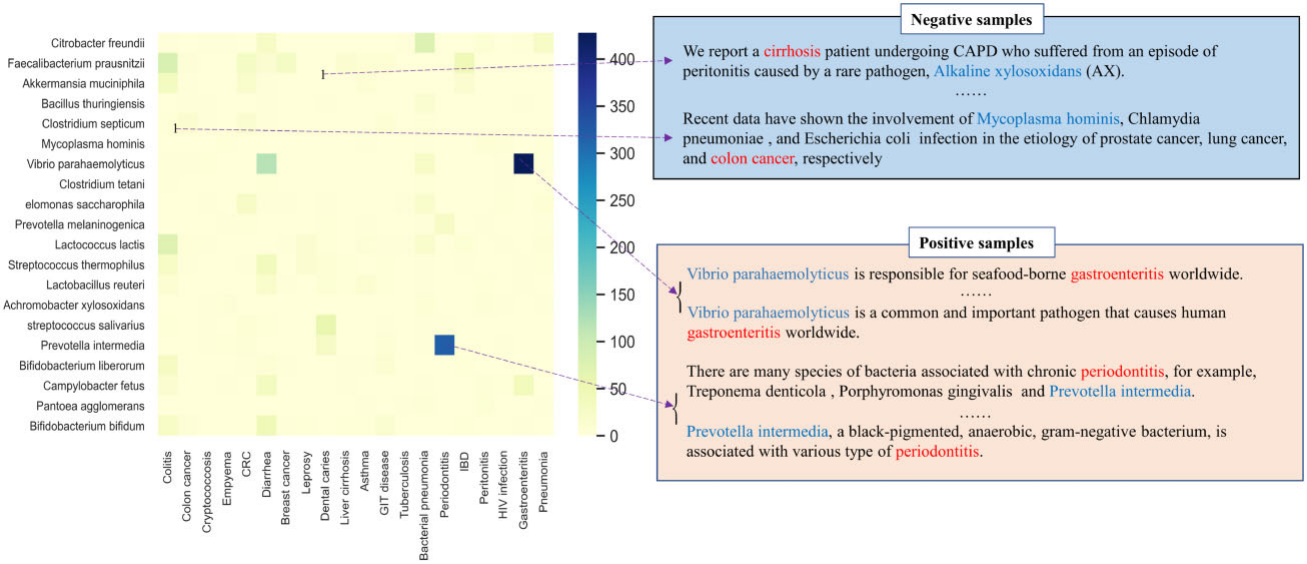
Example of unsupervised training data generation. The heatmap of the co-occurrence of 20 diseases and 20 microbes in the literature is shown in the left part of this figure. Gastroenteritis and Vibrio parahaemolyticus co-occur most frequently: more than 400 times, greater than a predefined threshold, so they are considered related and the corresponding sentences in the articles were selected as positive samples. On the contrary, the low co-occurrence couples, e.g. Liver Cirrhosis and Alkaline xylosoxidans, tend to be irrelevant and the corresponding sentences formed the negative samples

To generate the training data, a co-occurrence frequency matrix of the bioentities from sentences was first constructed from free-text in PubMed and PubMed Central. We chose entity pairs with the most co-occurrence counts and used two thresholds, ‘minimum co-occurrences *t*_1_’ and ‘truncating quantity *t*_2_’, to generate the positive data. Particularly, sentences containing a pair of entities co-occurring $\geq t_{1}$ times were considered. At most *t*_2_ of these sentences were retained to prevent the bias toward high frequency entity pairs. The default values of *t*_1_ = 10 and *t*_2_ = 50 were empirically set and used in all current experiments. To generate negative data, sentences containing entity pairs with the frequency of one in the matrix were included except the ones that contain a single disease term and biomarker term; because we found that the latter was more likely to be a positive case. Note that the negative sample means no direct association between two biomedical entities in a sentence. Same as co-occurrence-based methods, some rarer and possibly more relevant biomedical associations may be missed by ignoring low-occurring data. However, the samples generated here were used as the labeled data to train a model rather than used as the final result. The associative relation between a pair of bioentities is extracted by the trained model regardless of their co-occurrence frequency in the actual prediction stage. An example of the positive and negative data generation process is given in Figure 2. The complete training data was generated subject to a 6:4 ratio for positive and negative instances. The ratio is consistent with the fraction of positive and negative instances observed in the literature. The generated training data were further divided by an 8:2 ratio into training and validation sets for model parameter optimization on F1-score. The model performance was measured on independent datasets.

### 2.3 Entity extraction

Entity extraction is a pre-requisite step of relation extraction. The entities of interest were curated in term lists in advance. Currently, the following entity term lists have been curated—disease, microbiome, metabolite and gene as detailed in Supplementary Section S2. First, spaCy (Neumann *et al.*, 2019) was used for sentence splitting and tokenization. Then, similar to CIViCmine, exact string matching was applied to the tokenized sentences to extract entities. To achieve this, we first constructed a trie on all synonyms and then located the most extended term in sentences by traversing the trie. This strategy has a run time complexity of O(*n*) for a length-*n* sentence.

To improve the accuracy of entity recognition, rule-based filtering was further applied. If a disease term had a prefix of a letter followed by a dot, like ‘s. pneumonia’, the term was disregarded; we also removed term with a length less than four characters unless it was determined to be an abbreviation, conforming the pattern of ‘synonym + (entity)’.

### 2.4 Relation extraction from tables

**Fig. 3.**
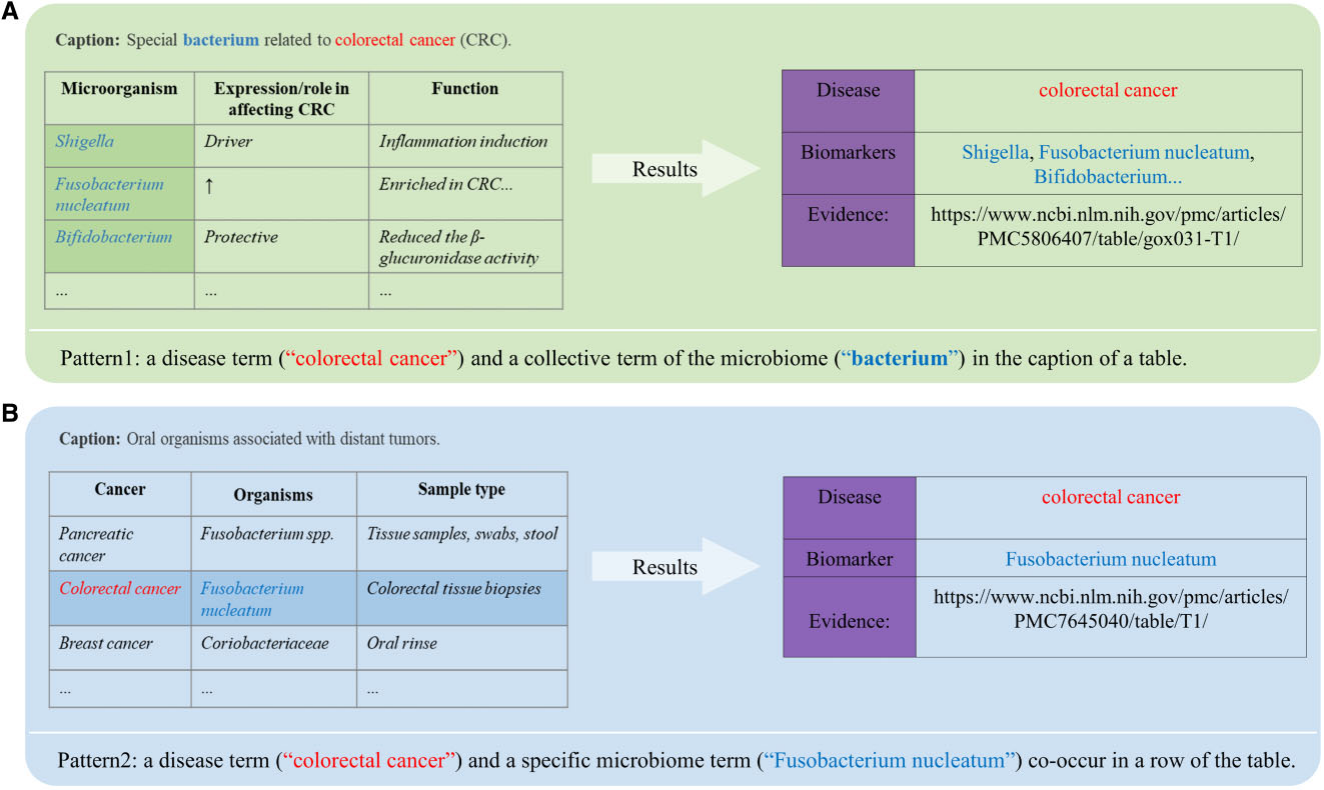
Illustration of relation extraction from tables. (**A**) CRC and a collective terms of the microbiome (‘bacterium’) co-occur in the caption of the table, so all microbes in the table body are considered to be related to CRC. (**B**) CRC and a specific microbiome term (‘Fusobacterium nucleatum’) co-occur in a row of the table. They are considered as related

Different from the classification-based relation extraction used for text, we used rule-based methods on tables. A table was first extracted and stored as a tuple (caption, table – head, table – body: list of data rows). The bioentity relations generally appear in two different patterns in a table as illustrated by the disease–microbiome relation extraction example: when a disease term and a collective term of the microbiome (e.g. ‘microbiome’, ‘bacteria’) co-occurred in the caption of a table and the microbiome terms were present in the body of the table, all microbiome terms in the table body were considered to be related to the disease (Fig. 3A). When a disease term and a specific microbiome term co-occurred in a row or caption of the table, they were considered to be related (Fig. 3B).

### 2.5 Granular relation classification between a disease and bioentities

**Fig. 4.**
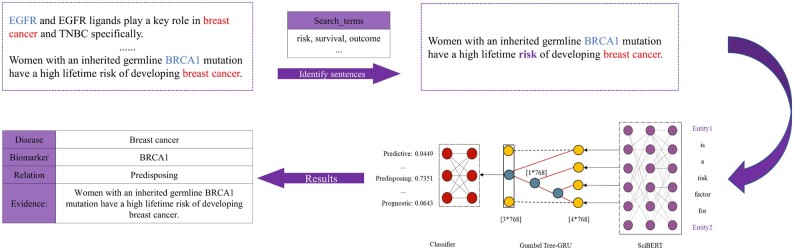
The workflow of MarkerGenie for classifying the granular relation types between a disease and bio-entities. When a disease and a bio-entity are determined by binary classification to be associative, MarkerGenie judges if there is a potential specific relation between them by using CIViCmine’s search terms. Then, the trained model is applied to predict the granular relation types (Predictive, Prognostic, Diagnostic, Predisposing or Treatment) of the filtered sentences

When a disease and a bioentity were determined by binary classification to be associative, MarkerGenie can further predict them to be one of the five granular relation types—*Predictive, Prognostic, Diagnostic, Predisposingor Treatment* if there is a potential specific relation between them judged by CIViCmine’s search terms (Fig. 4). The training sentences of this classification task were first generated via distant supervision method using knowledge databases of CBD (Zhang *et al.*, 2018), MarkerDB (Wishart *et al.*, 2021) and Oncomx (Dingerdissen *et al.*, 2020). Then we used a term list (e.g. ‘risk’ and ‘survival’) provided by CIViCmine to screen sentences that potentially contain one of the five specific relations. In addition, we expanded the term list by using pre-trained word vectors to include synonyms to increase the size of training data.

## 3. Results

In this section, we first demonstrate the improved accuracy of SBGT model by applying it on the curated benchmark datasets that were used by previous methods—the binary relation classification of PPI (Pyysalo *et al.*, 2007) and the multi-relation classification of drug–drug interaction (DDI’13) (Herrero-Zazo *et al.*, 2013). Next, we demonstrate the validity of automatic training data generation by applying MarkerGeine to disease–biomarker binary associative relation classification. This task does not require any prior knowledge or curated databases. However, when curated databases are available, MarkerGenie would generate training data via distant supervision strategy and produce multi-relation classification. This was demonstrated on disease–gene multi-relation extraction task as carried out in CIViCmine (Lever *et al.*, 2019). Finally, we demonstrate how MarkerGenie can aid biomarker discovery with a few case studies.

### 3.1 Binary relation classification

The SBGT model was first validated on the PPI corpora (Pyysalo *et al.*, 2007), which was used as a benchmark dataset by prior methods. The dataset information and hyper-parameters of SBGT are summarized in Table 1. To ensure the generalization of the learned model, we replaced the pair of proteins in each sentence with ‘PROTEIN1’ and ‘PROTEIN2’. In addition, all sentences were truncated or padded to a maximum length of 100. The performance of SBGT was compared with seven other state-of-the-art models—sdpCNN (Hua and Quan, 2016), sdpLSTM (Xu *et al.*, 2015), Bert (Devlin *et al.*, 2019), BioBERT (Lee *et al.*, 2020), DRCNN (Zhang *et al.*, 2019), Bi-LSTM (Hsieh *et al.*, 2017) and BioKGLM (Fei *et al.*, 2021). The evaluation scheme and parameters of the compared algorithms were all set per the original papers. The F1-scores of these methods are given in Table 2, where SBGT achieved 3.2% improvement over the runner up. Since some of the methods were evaluated with macro F1-score in the corresponding references, we also included this metric in Table 2, where SBGT showed consistent superiority to the compared models, including DCNN (Choi, 2018), Att-sdpLSTM (Yadav *et al.*, 2019), tLSTM (Ahmed *et al.*, 2019) and DRCNN.

**Table 1.** PPI and DDI’13 dataset information and hyper-parameters of SBGT

Dataset	PPI	DDI’13
No. classes	2	5
No. samples	9666	15 861
Evaluation scheme	10-fold cross-validation	77% training, 23% test
SBGT miniBatch-size	16	32
SBGT learning rate	1e−5	3e−5
SBGT num of epoch	50	10
SBGT optimizer	AdamW	AdamW

*Note*: The evaluation schemes were selected to be consistent with the methods under comparison.

**Table 2.** Comparison of SBGT and other methods on PPI dataset in terms of F1 score and macro-F1 score

Method	F1 score (%)	Method	Macro-F1 scores (%)
sdpCNN	75.2	DCNN	74.7
sdpLSTM	77.3	Att-sdpLSTM	81.7
Bert	82.3	tLSTM	89.1
BioBERT	84.6	DRCNN	91.1
DRCNN	86.9	—	—
Bi-LSTM	87.2	—	—
BioKGLM	89.3	—	—
**SBGT**	**92.4**	**SBGT**	**94.9**

### 3.2 Multi-relation classification

We applied SBGT to the DDI’13 dataset (Herrero-Zazo *et al.*, 2013) where the goal was to determine specific relations (defined as {NA, ADVICE, EFFECT, MECHANISM, INT}) given two drugs. Like binary classification, we replaced the pair of drugs in each sentence with ‘<ent1 >’ and ‘<ent2 >’ and all sentences were truncated or padded to a maximum length of 100. On this dataset, SBGT were trained with the hyper-parameters shown in Table 1. SBGT was compared with the seven other state-of-the-art models, including SCNN (Zhao *et al.*, 2016), CNN-bioWE (Liu *et al.*, 2016), MCCNN (Quan *et al.*, 2016), Joint AB-LSTM (Sahu and Anand, 2018), RvNN (Lim *et al.*, 2018), Position-aware LSTM (Zhou *et al.*, 2018) and BERE (Hong *et al.*, 2020) in terms of precision, recall and F1-score. As shown in Table 3, SBGT attained the best trade-off of precision and recall. In terms of F1 score, SBGT obtained a score of 77.1% that is ∼3% higher than that of the second best model.

**Table 3.** Comparison of SBGT and other methods on DDI’13 dataset in terms of Precision, Recall and F1 score

Method	P (%)	R (%)	F (%)
SCNN	69.1	65.1	67.0
CNN-bioWE	75.7	64.7	69.8
MCCNN	75.9	65.2	70.2
Joint AB-LSTM	73.4	69.6	71.5
RvNN	74.4	69.3	71.7
PM-BLSTM	75.8	70.4	73.0
BERE	76.9	71.3	73.9
**SBGT**	**80.3**	**74.2**	**77.1**

### 3.3 Disease–biomarker associative binary classification with automatic training data generation

We selected three major biomarker types—microbiome, metabolite and gene—to study their *associative* relations with diseases from publicly available articles of PubMed and PubMed Central. The labeled training data for these tasks are scarce or even missing though some have been manually curated (Lever *et al.*, 2019; Liu *et al.*, 2015). We introduced an unsupervised method that can automatically generate the labeled training data in Section 2.

**Fig. 5.**
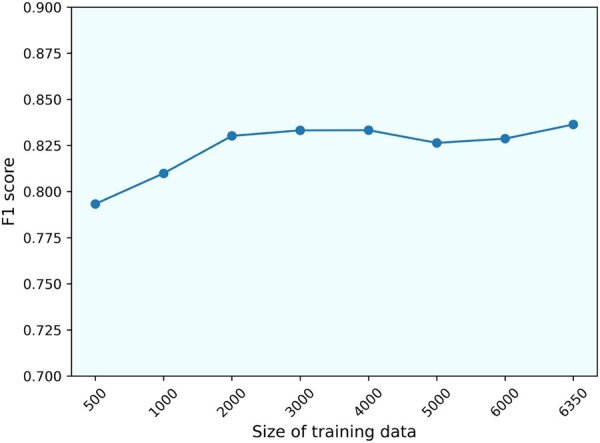
An illustration of the impact of automatically acquired training data on model performance. The SBGT model was trained on different sizes of disease–microbiome training dataset and F1 scores were obtained on the independent 477 test samples. Each experiment was repeated three times and the F1 score was the average

Admittedly, the automatic label generation can include many false positive instances—upon manual inspection, around 15–20% of the positive samples are incorrectly labeled. Yet, we can obtain a large amount of data within a few hours’ run time. We have obtained around 6000 disease–microbiome and 10 000 disease–metabolite or disease–gene training samples. The data of this size would be more suitable for deep learning strategies compared to the typical curated data size in the hundreds scale (Lever *et al.*, 2019). Though trained using noisy data increased the model bias, the overall model performance improved along with the size of training samples on the test data. As the example of disease–microbiome shown in Figure 5, the F1 value generally increased as more data became available.

**Fig. 6.**
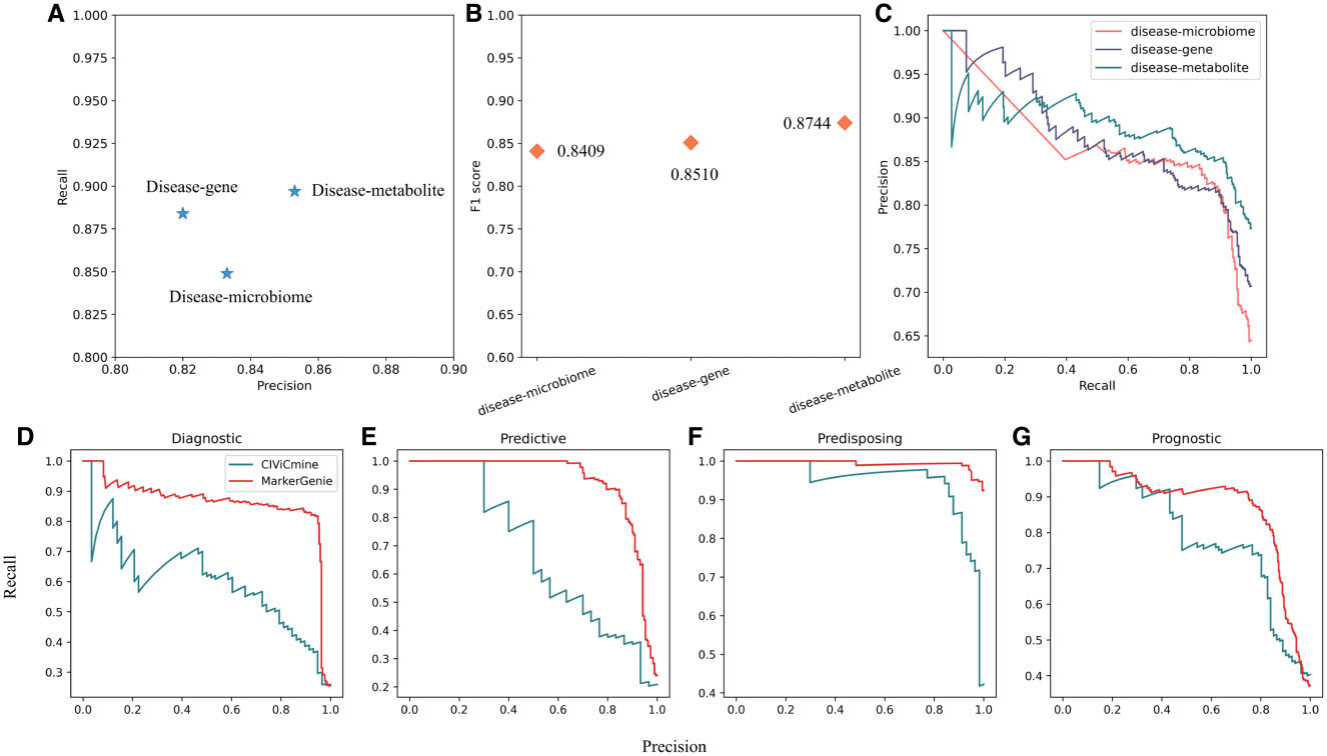
Performance of MarkerGenie on disease–biomarker relation identification. (**A**) Precision and recall of disease–biomarkers’ associative binary classification. (**B**) F1 scores of disease–biomarkers’ associative binary classification. (**C**) Precision-recall curves, due to the high threshold at the beginning, there are few samples marked as positive examples, so the upper left part of the curve fluctuates greatly. (**D–G**) Precision-recall curves of MarkerGenie and CIViCmine on four specific relation extraction, i.e. predictive, prognostic, diagnostic and predisposing

To evaluate the performance of MarkerGenie on the above tasks, we manually curated 477 disease–microbiome samples and 610 annotated disease–metabolite samples. For disease–gene prediction, 382 labeled disease–gene samples were directly obtained from Liu *et al.* (2015). MarkerGenie predicted disease–microbiome, disease–metabolite and disease–gene relations with precisions of 83.28%, 85.26% and 82.01%, respectively (Fig. 6A, the corresponding F1 scores and precision–recall curves are shown in Fig. 6B and C). Empirically, around 60–70% instances of disease–biomarker pairs co-occurring in the same sentence have a true positive relation, MarkerGenie therefore removed over 10–20% of the false positive instances. For these three tasks, MarkerGenie recalled 84.92%, 89.73% and 88.43% of the relations, respectively. We note that, the reported performance from Liu *et al.* (2015) on this disease–gene relation dataset obtained from the same study had both higher precision (∼5%) and recall (∼2%), yet its generalizability cannot be independently evaluated. Also note that, Liu *et al.* (2015) used the rule-based method that factors in the prior knowledge of validated disease–gene relations, which is generally unknown to the model.

### 3.4 Granular relation extraction via distant supervision

Following binary associative relation prediction, MarkerGenie can rely on disease–biomarker relation knowledge-bases to automatically generate training data via distant supervision, then yield more deterministic relation predictions. In this part, the performance of MarkerGenie was verified with the 250 test samples from Lever *et al.* (2019) that contains four granular relation types, *Diagnostic, Predictive, Predisposing* and *Prognostic* between cancers and genes. MarkerGenie was compared with CIViCmine (Lever *et al.*, 2019) in terms of precision and recall, where the precision–recall curves of the two methods are shown in Figure 6D–G. MarkerGenie obtained better precision and recall than CIViCmine.

In the following, we demonstrate how MarkerGenie can be applied to biomarker discoveries with different case studies.

#### 3.4.1 Identification of colorectal cancer-related microbes

Colorectal cancer (CRC) is the third most common cancer worldwide and one of the primary causes of cancer-related deaths (Rawla *et al.*, 2019). The association between CRC and the human gut microbiome is a focus of the current CRC research (Abdulla *et al.*, 2021; Chattopadhyay *et al.*, 2021; Sánchez-Alcoholado *et al.*, 2020). In this study, we used MarkerGenie to find the microbes related to CRC from the literature and manually verified the results.

**Fig. 7.**
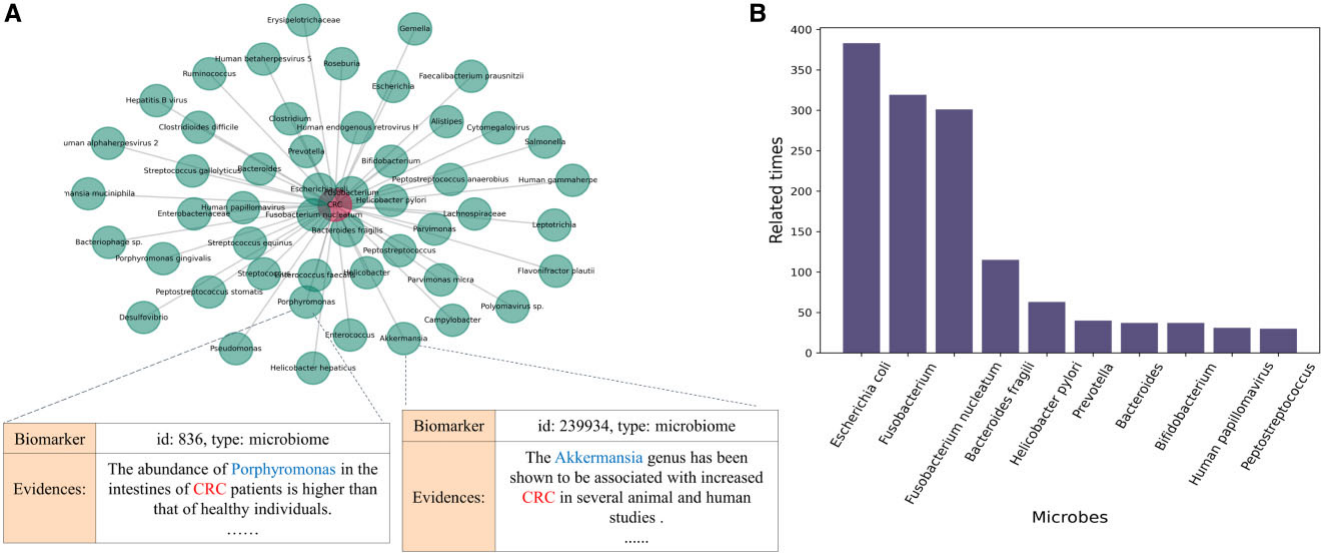
Statistics of microbes related to CRC returned by MarkerGenie. (**A**) Example of microbes and the corresponding sentences. (**B**) The top 10 microbes returned by MarkerGenie

In searching for microbes related to CRC, MarkerGenie returned a total of 2257 sentences that included 264 microbes. Among these 2257 sentences, 2118 were correctly predicted, whereas 98 were wrongly predicted and 41 were difficult to judge via manual inspection. The overall sentence’s binary classification precision is 93.8%. For microbes, 247 out of 264 microbes are associated with CRC. In Figure 7, an example list of microbes and the corresponding sentences is shown in A. The top 10 microbes with the highest occurrences are shown in B, among these, eight of them have been previously shown to be significantly associated with CRC in the meta-analysis study (Thomas *et al.*, 2019). The remaining two microbes ‘Helicobacter pylori’ and ‘Human papillomavirus’ also have been shown to be strongly related to CRC in more recent work (Chao *et al.*, 2020; Wang *et al.*, 2021). These results should provide a good reference to researchers studying CRC and the microbiome.

As discussed earlier, upon a positive prediction of binary associative relation between CRC and a microbe, we can further generate more deterministic relation classification via distant supervision (see Section 2.5). Here, MarkerGenie produced 185 predisposing, 181 predictive, 154 prognostic, 67 treatment and 33 diagnostic relations.

#### 3.4.2 Identification of breast cancer-related genes

**Fig. 8.**
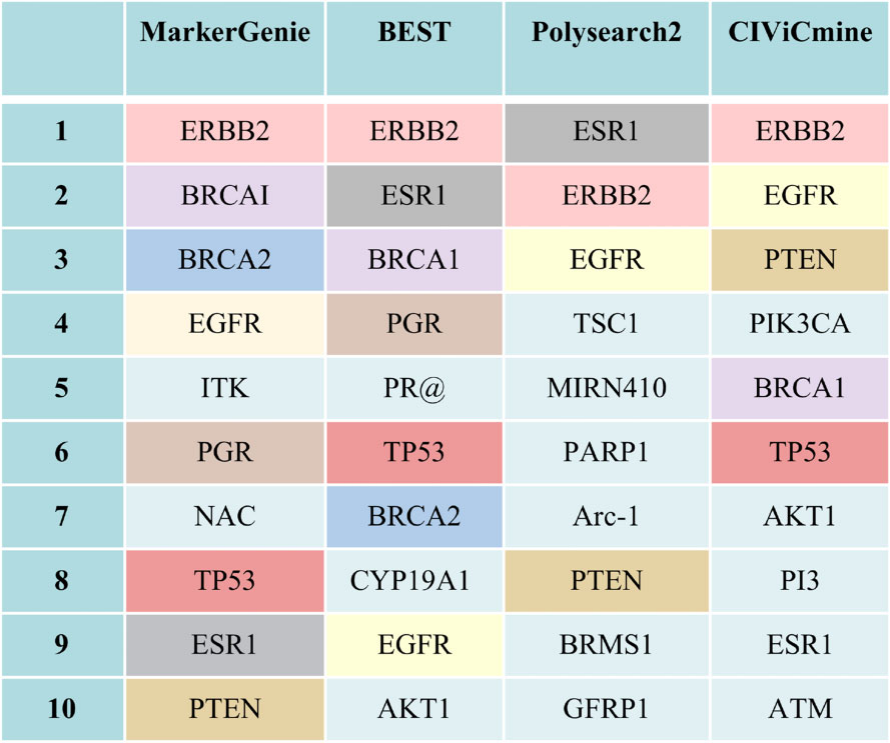
Top 10 genes retrieved with the query ‘Breast cancer’ by different systems

Identifying relevant genes is valuable for the early diagnosis, prevention and treatment of breast cancer (Kazmi *et al.*, 2022; Schettini *et al.*, 2021; Zhang *et al.*, 2022). We used MarkerGenie to search and rank the importance of the genes associated with breast cancer. Similar to BEST (Lee *et al.*, 2016), we presented the top 10 genes found in MarkerGenie along with the ones identified by BEST, Polysearch2 and CIViCmine in Figure 8. Eight of them were identified in at least one of the other methods and reported to be associated with breast cancer in CIViC knowledge-base (https://civicdb.org) or NCBI’s GENE database (https://www.ncbi.nlm.nih.gov/gene/). The remaining two genes ‘*ITK*’ and ‘*NAC*’ were false positives upon inspection. Specifically, the term ‘NAC’ refers to a type of therapy for breast cancer. For ‘ITK’, the term identified in association with breast cancer is ‘EMT’, which is an alias of ‘*ITK*’ gene. However, ‘EMT’ refers to ‘epithelial-mesenchymal transition’ that is a process linked to breast cancer. Both false positives are valid entries in the gene list but had different meanings in the text. To further improve accuracy, ambiguities of terms in the list need to be resolved.

#### 3.4.3 Disease–miRNA association inference

The output of MarkerGenie can also be directly used for other applications such as association prediction. We select the disease–miRNA association inference as a suitable application as it involves three-way interactions—disease–disease, disease–miRNA and miRNA–miRNA. The details of the inference method and experimental results are provided in Supplementary Section S3. Based on miRNA–miRNA functional similarity, disease–disease semantic similarity and the disease–miRNA associations identified by MarkerGenie, we can infer unknown disease–miRNA associations as accurately as the methods based on curated databases like HMDD (Huang *et al.*, 2019). MarkerGenie can serve as a surrogate for the laboriously curated databases.

## 4 Conclusions

In this work, we proposed a text-mining system, MarkerGenie, to identify bioentity relations from texts and tables of publications in PubMed and PubMed Central. The identification problem was formulated as a relation classification task. A new unsupervised training data generation method and new classification model SBGT were introduced and tested with benchmark datasets and real-world case studies. The experimental results demonstrated the effectiveness of the system. There are further rooms for improvement, including cross-sentence relations extraction, improving negative samples selection, and better ways to handle ambiguities of short entity terms such as gene symbols. It is also favorable to recognize the context (e.g. conditions of experiments and biology relevance) in which the biomarkers are identified and to improve the entity extraction with text-mining methods (e.g. PubTator and NER models).

## Supplementary Material

vbac035_Supplementary_Data
