# Supplementary material for: MarkerGenie: an NLP-enabled text-mining system for biomedical entity relation extraction
Source: Bioinform Adv. 2022 May 13;2(1):vbac035. doi: 10.1093/bioadv/vbac035 (PMC9710573; doi:10.1093/bioadv/vbac035)
Supplement: vbac035_Supplementary_Data [file vbac035_supplementary_data.pdf]

Supplementary Materials

S1 Implementation of MarkerGenie

S1.1 Corpus and index

MarkerGenie runs on more than 20 million PubMed abstracts and about 4 million PubMed Central full-text articles. All publications were downloaded and then indexed using elasticsearch (`http://www.elasticsearch.org`) to enable fast retrieval. The corpus and index are updated daily to contain the latest published articles.

S1.2 Front and back ends of MarkerGenie

The front-end interface of MarkerGenie is based on VUE, and the back-end framework is based on Django. There is an input text box on the homepage of MarkerGenie, where users can input the disease they want to query. Considering the diversity of user input and the fact that we ignored some general diseases, MarkerGenie performs fuzzy matching on user input. Fuzzy matching means that once a term in the term lists contains the keyword entered by the user instead of being precisely the same as the keyword, MarkerGenie will think that the word may be what the user wants to search. By doing so, users can even enter a body part to retrieve related diseases.

Taking the assumption that a user enters a disease and wants to find relevant biomarkers from the text of the publication, after the user selects the disease of interest and the type of biomarker (default is all biomarkers), MarkerGenie will output relevant biomarkers as well as the corresponding sentences. This process will be divided into five steps:

1. Check whether the disease and biomarker type have been searched in the search history table. If so, go to step 5.
2. Query the term lists to get all the synonyms of this disease, use elasticsearch to filter corpus.
3. Apply the exact string matching to annotate sentences, select those sentences containing both the disease and biomarkers.
4. Use the trained models to determine whether these sentences are discussing the relations between the disease and biomarkers. Keep associated sentences and write them into the search history table.
5. Find records in the search history table according to the selected disease and biomarker types and sort them in descending order according to the number of sentences discussing their relations.

At last, when users select a specific sentence, MarkerGenie will use elasticsearch to retrieve the article based on the article's id where the sentence is located. So the users can see more information, including the title of the article, abstract, time of publication, and links to Pubmed, Pubmed Center.

Also, some users may want to query the biomarker related to the disease of interest summarized in the table. Then, they only need to click the "table's ranking result" button. MarkerGenie will return biomarkers related to the disease entered by the user in the table and sorted by the tables' counts. Users can view the tables' specific content and the articles by clicking the "Detail" button. Some front pages of MarkerGenie are shown in Figure S1.

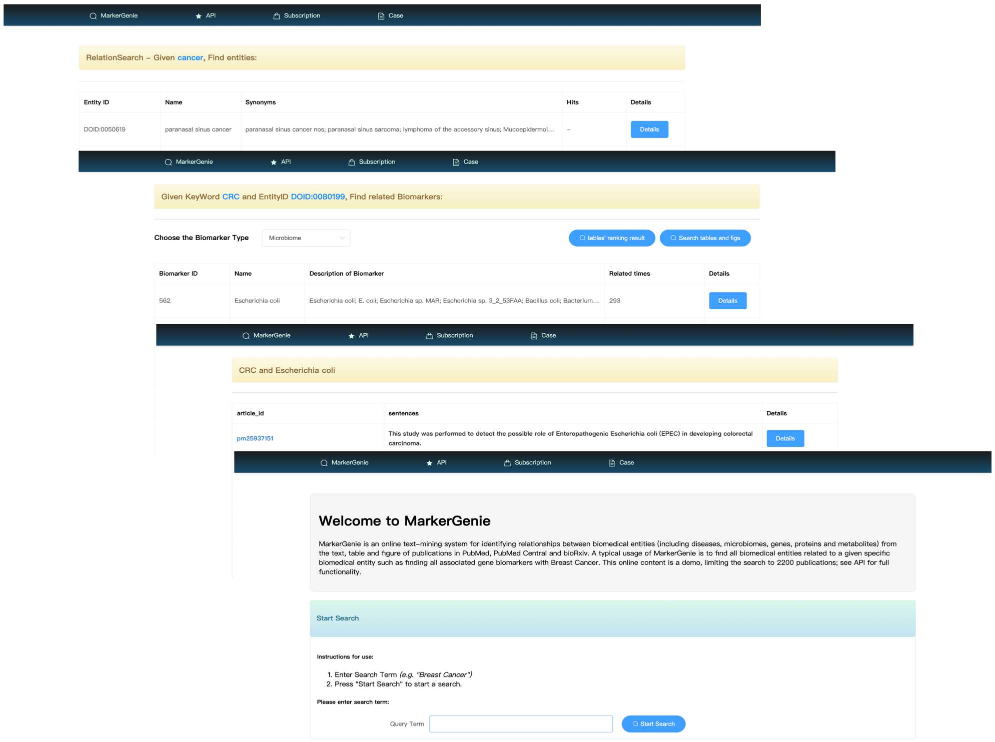

Figure S1: Front pages of MarkerGenie

S1.3 Integrating users' feedback

In order to improve the users' experience, MarkerGenie also integrates the function of collecting users' feedback. Users can give feedback whether our results are correct or not, these feedback will be used to fine-tune the model to improve the accuracy of predictions and provide users with results that are more in line with their expectations.

S1.4 Time performance

Time performance is another essential indicator that affects usage. The time spent by MarkerGenie is mainly divided into three parts:

1. Filter corpus from all publications based on the queried bioentities' synonyms.
2. Select sentences through exact string matching.
3. Apply the trained models to predict whether the sentences are positive or not.

The time performance of each part is shown in Table S1. In actual deployment, MarkerGenie speeds up by using a more lightweight model (Bi-GRU Cho *et al.* (2014)) and processing the last two parts in parallel by 20 processes. We did a series of speed tests to calculate the time spent in a search. As a result, a typical search with 2000 relevant abstracts and 200 full texts was finished in less than 30s. For a complete search containing about 100,000 abstracts and 100,000 full texts, the entire process took 30 minutes.

S2 Term lists of biomedical entities

Disease list was built from Disease Ontology (Schriml *et al.*, 2012) – diseases under the branch of "disease" (DOID: 4) in OBO Tree (disease classification based on etiology) were chosen, and the leaf nodes were in-

cluded as part of the synonyms of their parent node to limit the total number of disease types. Meanwhile, we removed genertic disease term such as "primary bacterial infectious disease". Next, the "Name" field of each disease was selected to be the unique name, the "DOID" was considered as its unique ID, and all "UMLS_CUI" in Xrefs were searched in UMLS Metathesaurus (Bodenreider, 2004) to find the synonyms.

Microbiome list was built from NCBI microbial taxonomy (Schoch *et al.*, 2020) - the taxonomy file "nodes.dmp" and "division.dmp" were used to obtain "division name" and "rank" columns, which were applied to filter for Bacteria, Viruses, Phages on or below family level. The column "tax_id" was linked to "names.dmp" from which we obtained the common name of the entity and its synonyms.

Metabolite list was built from HMDB (Wishart *et al.*, 2018) – from "metabolites.xml" (https://hmdb.ca/), we obtained the hmdb_id, common name and synonyms.

Gene list was curated from HGNG (Braschi *et al.*, 2019) by mapping Hugo GeneIDs to UMLS to get the synonyms.

Above term lists were inconsistently formatted and contained ambiguity and errors. We came up a list of rules to improve its accuracy:

1. Ignore those synonyms that are included in other synonyms.
2. If we want to take case into consideration, we add the lowercase format of the terms whose beginning is uppercase to synonyms.
3. Filter out terms with length less than 3.
4. Filter out terms with a comma, such as "Carcinoma, Colorectal".
5. Remove "(*)" in terms, such as "carcinoma of large intestine (diagnosis)".
6. Ignore the authority name of the microbiome cause this name is redundant.
7. Remove any microbes that are specified as "type material".
8. If a synonym ends with "gene", we add the part without "gene" to synonyms.
9. Replace "–" with a space, such as "pharynx–tumors".
10. Filter out terms with a semicolon such as "Carcinoma;bowel;large".

These term lists are updated every six months to include new biomedical entities.

S3 Disease-miRNA association inference

In this section, we demonstrate the application of the output of MarkerGenie: disease-miRNA association inference. This is based on the prior knowledge that miRNAs with similar functions are more likely associated with similar diseases, and diseases with high semantic similarity are more likely associated with similar miRNAs with similar functions (Wang *et al.*, 2010; You *et al.*, 2017).

	**Time of filtering corpus**	**Speed of filtering sentences**	**Speed of classifying sentences**
Pubmed	1-1.5 s/2000 abstracts	70 abstracts/s	80 sentences/s
PMC	10 s/2000 articles	30 articles/s	80 sentences/s

Table S1: Time performance of MarkerGenie's each part

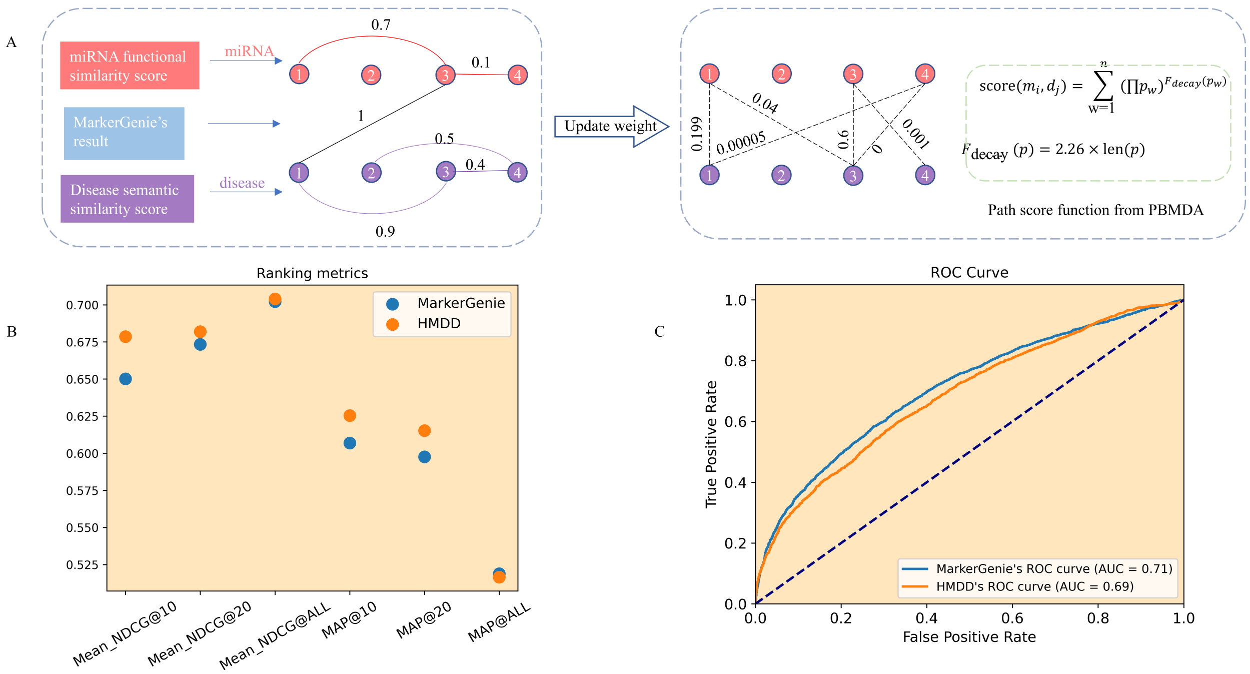

Figure S2: Illustration of disease-miRNA association inference and the corresponding results. **(A)** The process of constructing and updating disease-miRNA's graph. In the left part, the weight between diseases and the weight between miRNAs are initialized by the disease semantic similarity score matrix and miRNA functional similarity score matrix provided by PBMDA You *et al.* (2017), respectively. The weight between diseases and miRNAs is initialized according to MarkerGenie's result. Then, the path score function used by PBMDA was used to update the graph and some new disease-miRNA associations are shown in the right part. **(B)** Comparison of miRNA ranking based on MarkerGenie and HMDD in terms of mean NDCG and MAP. @10, @20, and @all indicate only the top 10, 20 and all miRNAs, respectively, are considered. **(C)** ROC curves of related-unrelated classification of miRNAs based on MarkerGenie and HMDD.

There are two major databases HMDD (Li *et al.*, 2014) and dbDEMC (Yang *et al.*, 2017) that have been used in prior studies. In HMDD (v2.0), there are a total of 5,430 experiment-supported miRNA-disease association entries that involve 383 diseases and 495 miRNAs. DbDEMC stores differentially expressed miRNAs in cancers detected by high-throughput or low-throughput methods. There are total of 56,647 miRNA-disease associations between 40 diseases and 4,495 miRNA in dbDEMC.

Given these databases of known disease-miRNA associations, a crucial task is to infer new associations (Xuan *et al.*, 2013), and PBMDA (You *et al.*, 2017) is one of the tools to achieve so. In brief, a graph is constructed, where each node represents an miRNA or a disease. An edge exists between each disease and each miRNA whenever an association is present in HMDD. A path-based computational model is built to predict missing edges, representing inferred associations.

Using the same graph based model, instead of building edges using HMDD database, we built edges and assigned edge weight from the output of MarkerGenie. We first obtained all MarkerGenie identified instances of disease-miRNA from the corpus, which contained 299 disease terms and 404 miRNAs overlapping with HMDD. A graph was built taking each miRNA or disease as a node, and the weight between diseases and the weight between miRNAs were set according to the disease semantic similarity score matrix and miRNA functional similarity score matrix provided by PBMDA (You *et al.*, 2017), respectively. The weight between diseases and miRNAs was initialized as follows: if disease i and miRNA j were predicted to be associative by MarkerGenie, the weight(i,j) was set to 1, otherwise 0. The same path score function used by PBMDA

was used to update the graph. (Figure S2A).

The inferred relations for both of the above methods were validated on relations among 31 diseases and 404 miRNAs that overlap with dbDEMC database, which is assumed to be independent of HMDD. An inferred relation is considered a true positive when it is present in the dbDEMC, otherwise, false positive. We adopted the two evaluation methods used in PBMDA. The first method used normalized discounted cumulative gain (NDCG) and mean average precision (MAP) (Radlinski and Craswell, 2010), considering top 10, 20 or all miRNAs. Higher scores indicate better inference. The second method used the ROC curve with respect to different edge weight cutoffs. As shown in Figure S2B and Figure S2C, the evaluation results have shown a comparable performance of MarkerGenie to HMDD. So, MarkerGenie's output can be used as a surrogate for the curated database.

References

Bodenreider, O. (2004). The unified medical language system (UMLS): integrating biomedical terminology. *Nucleic Acids Research*, **32**(suppl_1), D267–D270.

Braschi, B. *et al.* (2019). Genenames. org: the HGNC and VGNC resources in 2019. *Nucleic Acids Research*, **47**(D1), D786–D792.

Cho, K. *et al.* (2014). On the properties of neural machine translation: Encoder–decoder approaches. In *Proceedings of SSST-8, Eighth Workshop on Syntax, Semantics and Structure in Statistical Translation*, pages 103–111.

Li, Y. *et al.* (2014). HMDD v2.0 : a database for experimentally supported human microRNA and disease associations. *Nucleic Acids Research*, **42**(D1), D1070–D1074.

Radlinski, F. and Craswell, N. (2010). Comparing the sensitivity of information retrieval metrics. In *Proceedings of the 33rd International ACM SIGIR Conference on Research and Development in Information Retrieval*, pages 667–674.

Schoch, C. L. *et al.* (2020). NCBI Taxonomy: a comprehensive update on curation, resources and tools. *Database*, **1**, 21.

Schriml, L. M. *et al.* (2012). Disease ontology: a backbone for disease semantic integration. *Nucleic Acids Research*, **40**(D1), D940–D946.

Wang, D. *et al.* (2010). Inferring the human microRNA functional similarity and functional network based on microRNA-associated diseases. *Bioinformatics*, **26**(13), 1644–1650.

Wishart, D. S. *et al.* (2018). HMDB 4.0: the human metabolome database for 2018. *Nucleic Acids Research*, **46**(D1), D608–D617.

Xuan, P. *et al.* (2013). Prediction of microRNAs associated with human diseases based on weighted k most similar neighbors. *PLoS ONE*, **8**(8), e70204.

Yang, Z. *et al.* (2017). dbDEMC 2.0: updated database of differentially expressed mirnas in human cancers. *Nucleic Acids Research*, **45**(D1), D812–D818.

You, Z.-H. *et al.* (2017). PBMDA: A novel and effective path-based computational model for miRNA-disease association prediction. *PLoS Computational Biology*, **13**(3), e1005455.
